# The Effect of Caffeine and Trifluralin on Chromosome Doubling in Wheat Anther Culture

**DOI:** 10.3390/plants9010105

**Published:** 2020-01-15

**Authors:** Sue Broughton, Marieclaire Castello, Li Liu, Julie Killen, Anna Hepworth, Rebecca O’Leary

**Affiliations:** Department of Primary Industries and Regional Development, 3 Baron-Hay Court, South Perth 6151, Western Australia, Australia; Marieclaire.Castello@dpird.wa.gov.au (M.C.); Li.Liu@dpird.wa.gov.au (L.L.); Julie.Killen@dpird.wa.gov.au (J.K.); Anna.Hepworth@dpird.wa.gov.au (A.H.); Rebecca.O'Leary@dpird.wa.gov.au (R.O.)

**Keywords:** wheat doubled haploids, anther culture, chromosome doubling, caffeine, trifluralin

## Abstract

Challenges for wheat doubled haploid (DH) production using anther culture include genotype variability in green plant regeneration and spontaneous chromosome doubling. The frequency of chromosome doubling in our program can vary from 14% to 80%. Caffeine or trifluralin was applied at the start of the induction phase to improve early genome doubling. Caffeine treatment at 0.5 mM for 24 h significantly improved green plant production in two of the six spring wheat crosses but had no effect on the other crosses. The improvements were observed in Trojan/Havoc and Lancer/LPB14-0392, where green plant numbers increased by 14% and 27% to 161 and 42 green plants per 30 anthers, respectively. Caffeine had no significant effect on chromosome doubling, despite a higher frequency of doubling in several caffeine treatments in the first experiment (67–68%) compared to the control (56%). In contrast, trifluralin significantly improved doubling following a 48 h treatment, from 38% in the control to 51% and 53% in the 1 µM and 3 µM trifluralin treatments, respectively. However, trifluralin had a significant negative effect on green plant regeneration, declining from 31.8 green plants per 20 anthers (control) to 9–25 green plants per 20 anthers in the trifluralin treatments. Further work is required to identify a treatment regime with caffeine and/or anti-mitotic herbicides that consistently increases chromosome doubling in wheat without reducing green plant regeneration.

## 1. Introduction

The production of doubled haploid (DH) lines remains an important tool for the rapid generation of fixed lines for breeding and research purposes. In self-pollinating crops such as wheat, the time to variety release can be reduced by three to four years when DHs are used. Additionally, phenotypic evaluation and selection is more reliable and accurate in DH populations. The benefits and applications of DHs have been reviewed extensively, and the large numbers of wheat DH varieties attest to the success of the technology [1,2,3]. In addition to variety development, DH populations are a significant research tool for mapping single locus genes and QTL controlling traits of interest [4,5,6]. In their recent review of wheat DHs, Devaux and Cistué [3] also indicated the usefulness of DHs for their application in genome wide association studies (GWAS), genomic selection (GS) and sequencing activities. Doubled haploid technology can also be used in tandem with transformation and gene-editing technologies, where isolated microspores or microspore-derived embryos can be used as targets. For these technologies, the totipotent microspore is a valuable tool, and homozygous DH transgenic or gene-edited plants can be generated in a single step [7,8,9,10].

In Western Australia, large numbers of spring bread wheat (*Triticum aestivum* L.) DH lines are produced each year using anther culture. In 2018, approximately 15,000 wheat DH lines were produced. Improvements in wheat DH production have resulted from co-culture with ovaries [11] and *n*-butanol treatment [12,13]. Approximately 90% of the DH lines developed in our program are destined for plant breeding companies, with the remaining populations used for research projects throughout Australia [14,15]. Genotype variability in key production parameters, such as embryo production, green plant regeneration and chromosome doubling, is an ongoing challenge, especially for the high-throughput production of breeding populations.

We rely on spontaneous chromosome doubling to restore fertility. This avoids the necessity to screen regenerant plants for ploidy and treat haploid plants with colchicine using root dipping “immersion” treatments [16,17], and the associated issues of plant mortality, ploidy chimeras and variable seed set that can occur as a result of this treatment [18]. In microspore-derived wheat and barley DHs, nuclear fusion is widely accepted as the mechanism responsible for spontaneous chromosome doubling [19,20,21]. In barley, the frequency of spontaneous doubling is relatively high, 60% to 90% [22,23]. In wheat, however, the frequency of spontaneous doubling can vary more widely, with reports of 25% to 70% [23]. In our program, which predominantly handles Australian spring wheat crosses, we have observed frequencies from 14% to 80%.

Anti-mitotic chemicals, such as colchicine, can also be applied in vitro to promote chromosome doubling at an early stage. This can be labor- and cost-effective and avoid some of the problems of colchicine root dipping. The addition of colchicine to anther and microspore induction media has successfully improved doubling in wheat [18,24,25] as well as other species, including *Brassica napus* [26,27], triticale [28], rice [29] and red pepper [30]. Colchicine also has a positive effect on embryogenesis and can replace the heat pretreatment normally required for embryogenesis in *B. napus* [31]. Increases in embryo and/or green plant numbers following in vitro colchicine application have also been reported in wheat, although the effects varied with genotype [18,24,25]. While colchicine has been widely used to induce polyploidy in many plant species, it also has negative aspects. It has been shown to have a low affinity for plant microtubules and therefore must be used at relatively high concentrations [32,33]. It is also toxic to humans and has a high affinity for vertebrate microtubules [34].

Several herbicides also target mitosis as a primary mechanism of action. They belong to a range of chemically diverse classes, including dinitroanilines (trifluralin and oryzalin), phosphorothioamidates (aminoprophos-methyl or APM), benzamides (pronamide), carbamates (chlorpropham and isopropyl *N*-3-chlorophenyl carbamate) and others [34]. Studies on the mechanism of oryzalin and APM have shown they bind to tubulin proteins, inhibiting microtubule polymerization and promoting depolymerization of the anaphase spindle [35,36]. Mitosis and cell division are inhibited, and affected cells may contain polyploid nuclei. Like colchicine, these chemicals have been applied to a range of plant species to induce polyploidy [34]. Trifluralin, oryzalin and APM have also been used to induce chromosome doubling during androgenesis in wheat [37], *B. napus* [38,39,40], maize [41] and cork oak [42] and during parthenogenesis in beet [43] and cucumber [44]. Because these chemicals have a much higher affinity for plant microtubules than colchicine, they can be applied at micromolar concentrations [35,45]. Additionally, these chemicals do not bind to animal microtubules [35,36,45], reducing the toxicity risk to humans.

There is evidence that these chemicals can also stimulate embryogenesis, which is not surprising, given that their effects on plant microtubules are similar to colchicine. Trifluralin and APM stimulated embryogenesis in wheat microspores and oryzalin, trifluralin and APM stimulated embryogenesis in *B. napus* [37,38]. In these studies, the herbicides were applied at concentrations of 0.1–10 µM (wheat) or 0.3–30 µM (*B. napus*) for either 24 or 48 h. Stimulatory effects were observed at low concentrations (0.3–1.0 µM) of the herbicides, while higher concentrations inhibited embryo formation in both species. In contrast, plant fertility improved with increasing herbicide concentration. In wheat, the highest percentage of fertile plants was obtained with 10 µM trifluralin or APM applied for 48 h. Colchicine was also included in the *B. napus* study, although at much higher concentrations (3–3000 µM) and for shorter exposure times (6–24 h). The response curves for colchicine were similar to the herbicides, with small improvements in embryo numbers at low colchicine concentrations, while concentrations above 300 µM were considered toxic. Again, plant fertility increased with increasing colchicine concentration and exposure time. Data from these studies indicate that anti-mitotic herbicides such as oryzalin, trifluralin and APM can have similar in vitro effects to colchicine.

Exposing plant cells to caffeine can also result in binucleate and multi-nucleate cells by impeding cytokinesis. Although cell plate formation commences normally in the presence of caffeine, it is never completed [46,47,48]. The cell plate is synthesized by the phragmoplast, which expands centrifugally. Dynamic microtubules depolymerize in the central region, where cell plate synthesis is completed, and re-polymerize at the expanding phragmoplast front, where cell plate synthesis will next take place. Golgi-derived vesicles fuse into a continuous membrane network in the center of the phragmoplast, and callose forms a coat-like structure on the membrane surface, later replaced by cell wall polysaccharides [49,50]. In the presence of caffeine, however, both the deposition of callose and the redistribution of phragmoplast microtubules is completely inhibited, and the deposition of callose in the cell plate appears tightly related to the depolymerization of microtubules at the central region of the phragmoplast [49,51]. Although the exact mechanism of caffeine remains unclear, it has been proposed that Ca^2+^ gradients and the reduction of Ca^2+^ levels near the cell plate play a role [48,49,52].

There is a limited number of studies in which caffeine has been used to induce polyploidy for practical purposes. Espino and Vazquez [53] applied caffeine and colchicine to detached cultured leaves of African violet, *Saintpaulia ionantha*, to induce polyploidy; however, the frequency of polyploids following caffeine treatment was very low. Lim et al. [54] injected caffeine solution into the buds of interspecific Lilium crosses to restore gametic fertility to obtain 2n gametes. In wheat, caffeine has been tested as an alternative to colchicine to induce chromosome doubling in haploids generated from interspecific (wheat × maize) crosses [55]. In that study, caffeine was tested in immersion/root dipping treatments over a range of concentrations (0.3–10 g/L) and times (3–24 h). Various treatments resulted in pollen shedding and substantial seed set compared with the untreated haploid controls. To our knowledge, caffeine has not been tested as an in vitro doubling agent following androgenesis.

This study aimed to determine whether chemicals such as caffeine or trifluralin, applied in vitro during anther culture, could improve green plant regeneration and/or the frequency of chromosome doubling in wheat. The effects of caffeine and trifluralin on albino plant production were also considered.

## 2. Results

### 2.1. Experiment 1: The Effect of Caffeine on Green and Albino Plant Regeneration and Chromosome Doubling (One Cross)

Caffeine application for 48 h had a significant negative effect on green plant regeneration in the (Yr57/3*Gladius#60)/2*Trojan cross (*p* < 0.001). In both 48 h treatments, the number of green plants decreased significantly, from 38.6 green plants per 20 anthers in the control to 24.3 and 27.5 green plants in the 0.5 mM and 1.5 mM treatments, respectively (Table 1). The 24 h caffeine treatments had no effect on green plant production, despite higher green plant numbers following the 0.5 mM/24 h treatment. Caffeine treatments also affected albino plant regeneration (*p* < 0.001), both positively and negatively. The number of albino plants decreased significantly in the 1.5 mM/24 h treatment, from 16.6 (control) to 11.0 (caffeine), and increased significantly in the 0.5 mM/48 h treatment, to 22.0 albino plants per 20 anthers (Table 1).

The frequency of chromosome doubling was higher in three of the four caffeine treatments compared to the control, but the increases were not significant (*p* = 0.327) (Table 1). Despite the lack of significance, the results were encouraging, with three treatments resulting in 67–68% doubling, compared to 56% in the control. When green plant regeneration and chromosome doubling were combined in a success index, the 0.5 mM/24 h caffeine treatment was the most successful, with 29.5 DHs per 20 anthers compared with 20.1 in the control (Table 5). The increase warrants further investigation in a larger experiment (Experiment 2).

### 2.2. Experiment 2: The Effect of Caffeine on Green Plant Regeneration and Chromosome Doubling (Five Crosses)

Caffeine treatment resulted in significantly higher green plant regeneration in Trojan/Havoc (*p* < 0.001, full analysis) and Lancer/LPB14-0392 (*p* < 0.001, post-hoc analysis). In Trojan/Havoc, green plants per 30 anthers increased by 14%, from 141.1 in the control to 161.0 following caffeine treatment (Table 2). In Lancer/LPB14-0392, green plants per 30 anthers increased by 27%, from 32.8 (control) to 41.8 (caffeine). Caffeine had no significant effect on green plant numbers in the remaining three crosses (*p* = 0.672).

Genotype effects were significant in all analyses (*p* < 0.001), reflecting the variation in green plant regeneration between crosses. Green plant regeneration varied widely between the five crosses, ranging from 6 to 151 green plants per 30 anthers (approximately 12–302 green plants per spike) (Table 2).

Caffeine treatment did not significantly affect the frequency of chromosome doubling in any of the five crosses in this experiment (Table 3). The differences between treatments were small (5% or less) and not significant at either of the two grow-out locations, the Department of Primary Industries and Regional Development, South Perth (DPI) (*p* = 0.584) or Virginia, South Australia (VIR) (*p* = 0.245). The G*T interaction was also non-significant at both locations (*p* = 0.52 (DPI) and *p* = 0.868 (VIR)), indicating that the crosses responded in a similar manner. The frequency of chromosome doubling between crosses, however, varied significantly at both locations (*p* < 0.001), with genotype means ranging from 31% in Lancer/LPB14-0392 to 61% in Trojan/Havoc and Scepter/05PN240.

### 2.3. Experiment 3: The Effect of Trifluralin on Green and Albino Plant Regeneration and Chromosome Doubling

All trifluralin treatments had a significant negative effect on green plant regeneration in Tammarin Rock (*p* < 0.001). The control treatment yielded 31.8 green plants per 20 anthers (approximately 95 green plants per spike) compared with 9–25 green plants per 20 anthers in the trifluralin treatments (Table 4). When anthers were exposed to 1 µM trifluralin for 24 h, the number of green plants per 20 anthers decreased significantly, from 31.8 in the control treatment to 25.1 in the trifluralin treatment (21% reduction). Extending the exposure time to 48 h caused further significant reductions in green plant numbers, to 20.0 green plants per 20 anthers (37% reduction). Larger reductions in green plant numbers occurred when anthers were exposed to 3 µM trifluralin, declining from 31.8 to 10.8 green plants per 20 anthers (66% reduction) after 24 h exposure and from 9.2 green plants per 20 anthers (71% reduction) after 48 h. Trifluralin also had a significant effect on albino plant numbers (*p* < 0.001), with significantly more albino plants produced in the 1 µM/48 h treatment and significantly fewer albino plants produced in both the 3 µM treatments, compared to the control (Table 4).

In contrast to green plant regeneration, trifluralin had a significant positive effect on the frequency of chromosome doubling in Tammarin Rock (*p* < 0.10). All trifluralin treatments had higher rates of chromosome doubling than the control, with significant improvements over the control in both 48 h treatments (Table 4). In these treatments, doubling increased from 38% (control) to 51% and 53% in the 1 µM and 3 µM trifluralin treatments, respectively. Despite improved doubling frequencies following treatment with trifluralin, the reductions in green plant regeneration meant that the success index values (DHs per 20 anthers) for trifluralin treatments were either similar to or less than the control (Table 5).

## 3. Discussion

Exposing anthers to caffeine or trifluralin at the start of the induction phase yielded mixed results in this study. Caffeine treatment at 5 mM for 24 h significantly improved green plant production in two of the six spring wheat crosses but had no effect on the other four crosses. The improvements were observed in two responsive crosses, Trojan/Havoc and Lancer/LPB14-0392, where green plant numbers increased by 14% and 27% to 161 and 42 green plants per 30 anthers, respectively. Increasing the time anthers were exposed to caffeine from 24 to 48 h significantly reduced the number of green plants in Experiment 1, where only one cross was tested.

Caffeine had no significant effect on the frequency of chromosome doubling despite higher doubling in Experiment 1, from 56% in the control to 67–68% in three of the four caffeine treatments. When the results from Experiment 1 were expressed as a success index (DHs per 20 anthers), the 0.5 mM/24 h caffeine treatment yielded sufficient improvement to warrant further investigation, with 29.5 DHs per 20 anthers, compared with 20.1 in the control. However, when this treatment was tested on five crosses in Experiment 2, there was little difference between the control and caffeine treatment means (5% or less).

To our knowledge, caffeine has not been tested as an in vitro doubling agent in anther or microspore culture. Although caffeine did not significantly improve doubling in this study, it did result in chromosome doubling and restored fertility in wheat haploids when applied as immersion/root dipping treatments [55]. Caffeine may also promote embryogenesis, given that it can affect phragmoplast microtubules during cell division and cytokinesis [51] and its application resulted in modest improvements in green plant production in some genotypes in this study. It is widely accepted that the cytoskeleton is involved in reprogramming microspores toward androgenesis [1,20]. The disruption of spindle microtubules by colchicine and anti-mitotic herbicides has stimulated microspore embryogenesis in several species (see Section 1) and the disruption of cortical microtubules by *n*-butanol has stimulated embryogenesis in wheat [12,13].

In terms of this study, the concentration of caffeine may have been too low. The selected concentrations were based on previous studies with colchicine and caffeine. For example, colchicine is generally applied at 0.1% (*w*/*v*) (2.5 mM) for immersion/root dipping treatments in cereals [16,17] but at lower concentrations (0.3 to 1.0 mM) when applied in vitro to anther and microspore cultures [18,25]. When caffeine was tested in a series of immersion/root dipping treatments in wheat, 3 g/L (15.4 mM) for 24 h was the most successful treatment in terms of seed recovery and the size and incidence of fertile sectors [55]. To test the in vitro application of caffeine in this study, concentrations of 0.5 and 1.5 mM were selected. The fact that there were no significant improvements in doubling, however, indicates that higher concentrations of caffeine may be required. Anther walls may also act as a filter, preventing the absorption of caffeine. Soriano et al. [18] applied colchicine during anther and microspore culture to the wheat variety Pavon and obtained smaller improvements in doubling with anther culture compared to microspore culture. Based on the results of Pulido et al. [56], they proposed that the anther wall may act as a filter, preventing colchicine absorption. We may be seeing the same effect, especially as dimethyl sulfoxide (DMSO) was not included in our experiments with caffeine. Given the preliminary results from this study, it would be beneficial to test higher concentrations of caffeine for 24 h or less and include DMSO in the treatment. It would also be useful to test more than one genotype so that a more robust treatment can be identified.

In contrast to caffeine, trifluralin significantly decreased green plant regeneration and significantly increased chromosome doubling in the variety Tammarin Rock. The control treatment yielded 31.8 green plants per 20 anthers (approximately 95 green plants per spike), compared with 9–25 green plants per 20 anthers in the trifluralin treatments. Green plant numbers were reduced by 21% to 71% in the trifluralin treatments, with increasing concentration and exposure times resulting in stepwise significant reductions in the number of green plants. However, every trifluralin treatment had higher rates of chromosome doubling than the control, with significant improvements in both treatments where anthers were exposed to trifluralin for 48 h. In these treatments, doubling improved from 38% (control) to 51% and 53% in the 1 µM and 3 µM trifluralin treatments, respectively. Despite the improved doubling frequencies following trifluralin treatment, the reductions in green plants meant that the success indices for the trifluralin treatments were either similar to the control (~10–11 DH per 20 anthers) or less than the control (~5 DHs per 20 anthers).

The concentrations and exposure times of trifluralin selected for use in this study (1 and 3 µM) were based on the results of Hansen and Andersen [37], who tested both trifluralin and APM at concentrations ranging from 0.1 to 10 µM in a wheat microspore study. They observed that low concentrations could stimulate embryo production (relative to the treated controls) and plant regeneration, but higher concentrations reduced embryo and plant numbers. In contrast, the percentage of fertile diploid plants increased steadily with increasing concentrations of trifluralin or APM. When the results were combined in a success index (DHs per spike), the best results were obtained at concentrations between 1 and 3 µM.

In the present anther culture study, we did not observe any positive effects of trifluralin on green plant production, even with low concentrations (1 µM) of trifluralin. This might reflect the fact that we used anther culture and not microspore culture. In our experiment, the trifluralin (dissolved in DMSO) solution would have penetrated the anthers and remained in contact with microspores after the treatment finished. In contrast, Hansen and Andersen [37] rinsed their microspores following treatment with the herbicides. Given our results, it may be useful to try a rinse step, as well as more exposure time/concentration combinations. Additionally, we can test other culture phases such as embryos and alternative solvents such as acetone [34].

This study was a preliminary investigation into the application of in vitro doubling agents to improve the frequency of chromosome doubling following anther culture. Further work is required to identify a treatment regime with caffeine and/or anti-mitotic herbicides that consistently increases chromosome doubling but does not significantly reduce green plant production.

## 4. Materials and Methods

### 4.1. Germplasm and Donor Plant Growth

A range of spring wheat (*Triticum aestivum* L.) crosses and the variety, Tammarin Rock, was used in this study (Table 6). The F_1_ seed for all crosses was provided by LongReach Plant Breeders Management Pty Ltd. (Adelaide, Australia). Donor plant growth was as described in Broughton et al. [57] with plants grown in controlled environment rooms at 18/13 °C (day/night) with a 12 h photoperiod.

### 4.2. Experiment 1. The Effect of Caffeine on Green and Albino Plant Regeneration and Chromosome Doubling (One Cross)

#### 4.2.1. Anther Culture

Twenty spikes were harvested from three F1 donor plants of the cross, (Yr57/3*Gladius#60)/2*Trojan (6–7 spikes per plant). The spikes were harvested over 4 days and stored at 4 °C for up to 6 days. Spikes were harvested when microspores were at the late uninucleate stage, although in some spikes, mitosis was visible. Microspore stage was determined by squashing anthers in 2% acetocarmine stain (*w*/*v*) and examining the microspores under 400× magnification. All spikes were sterilized and processed on the same day using the protocol described in Broughton et al. [57]. The media used were (1) solid 1.0 M mannitol pretreatment medium, (2) liquid induction medium (LIM) and (3) solid regeneration medium, as described in Broughton et al. [57] with iron-source ethylenediaminetetraacetic acid ferric sodium salt (FeNaEDTA). The anther culture protocol involved a number of steps, including (1) anther pretreatment to induce embryogenesis (mannitol (5 days) and *n*-butanol (5 h)); (2) an induction phase, in which anthers and ovaries were co-incubated in LIM for 4–5 weeks, and (3) a regeneration phase, in which plants were grown on solid regeneration medium prior to transfer to soil (4–5 weeks).

Following spike sterilization, 60 anthers were removed from each spike and placed on a 90 × 14 mm Petri dish containing mannitol pretreatment medium. Each 90 mm dish was divided into three sections by marking the base of the dish. The anthers were taken from five spikelets (10 florets) on each side of the spike, making 10 spikelets (20 florets) in total. Each floret contained three anthers, which were separated after removal from the floret and placed on a different section of the dish so they could later be allocated to the three treatments (20 anthers per spike/treatment combination). This design was used to ensure that anthers from each spike were evenly divided between the three treatments. Immediately following anther removal, 20 ovaries were removed from each spike and placed in two 55 × 14 mm Petri dishes containing 4 mL LIM (10 ovaries per dish). Additional spikes at the same stage of development were also processed for ovary removal, so there was a dish of ovaries available for each of the 60 sets of anthers. All dishes were sealed with Parafilm™ and incubated in the dark at 25 °C for 5 days.

Following the 5 day mannitol pretreatment, each of the three sets of 20 anthers (per spike) were transferred to three 55 × 14 mm Petri dishes containing 4 mL LIM and denoted “A”, “B” and “C”. When anthers from all spikes had been transferred, 8 µL *n*-butanol (0.2% v/v) was added to each dish (60 dishes in total). Dishes were covered with alfoil (but left unsealed) and incubated in the dark at 25 °C for 5 h. After 5 h, the LIM plus *n*-butanol solution was removed from each dish using a sterile pipette. The 20 dishes denoted “A” were treated as controls, and the standard protocol was applied with a dish of ovaries and ovary-conditioned LIM tipped into each dish of pre-treated anthers. If any ovaries remained in the dish and did not get tipped, they were gently transferred with forceps. Dishes were sealed with Parafilm™, and the anthers and ovaries were co-incubated in the dark at 25 °C for 4–5 weeks.

Fresh LIM (4 mL) was added to the anthers in the “B” and “C” dishes, and caffeine stock (0.5 mL) was added to each dish. Caffeine stocks were prepared by dissolving caffeine (C0750, Sigma-Aldrich, Sydney, Australia) in deionized water, followed by filter sterilization. Different concentrations of caffeine stocks were prepared, so the same amount of caffeine stock was added to the LIM for each treatment. For spikes 1–10, the final caffeine concentration was 0.5 mM, while for spikes 11–20, the caffeine concentration was 1.5 mM. The anthers were exposed to caffeine for either 24 (“B” dishes) or 48 h (“C” dishes) (Table 6). There were 10 dishes (replicates) for each spike/treatment combination. After 24 or 48 h, the caffeine plus LIM solution was removed, and a dish of ovaries and ovary-conditioned LIM was added to each dish of anthers. Dishes were sealed with Parafilm™, and the anthers and ovaries were co-incubated in the dark at 25 °C for 4–5 weeks.

#### 4.2.2. Plant Regeneration and Grow-Out

After 4–5 weeks, each dish of embryos (60) was tipped onto one dish of regeneration medium in a 90 × 20 mm Petri dish. Excess LIM was tipped off or removed by pipette. Dishes were sealed with Parafilm™ and incubated in the dark at 25 °C for 7 days, then transferred to a constant temperature room with a 12 h photoperiod (light/dark) at 25 °C for a further 7–14 days. Individual green plants were then sub-cultured to fresh regeneration medium (approximately 5–10 plants per dish) in 90 × 20 mm Petri dishes. At this stage, any albino plants were counted and discarded as well as any excess green plants (>10). The total number of green and albino plants for each treatment/spike combination was recorded. Dishes of sub-cultured green plants were returned to the constant temperature room for a further 2–3 weeks until root growth was sufficient, and the plants were ready to be transplanted to soil. Five green plants from each treatment/spike combination were transplanted to soil (50 plants per treatment). There were more plants transplanted from the control treatment (100), as a control was included in each set of 10 spikes. Plants were transplanted to soil at DPIRD greenhouses in South Perth and grown to maturity to obtain DH plants. Ploidy was determined visually, and plants that set seed were classed as DHs while sterile plants were classed as haploids.

### 4.3. Experiment 2. The Effect of Caffeine on Green Plant Regeneration and Chromosome Doubling (Five Crosses)

#### 4.3.1. Anther Culture

The 0.5 mM/24 h caffeine treatment was selected for further evaluation of five crosses that were being processed for DH production (Table 7). Spikes from F1 donor plants were harvested over 5 days and stored at 4 °C for up to 3 days.

The anther culture protocol was the same as Experiment 1, but the design was simplified to two treatments: control and caffeine (0.5 mM/24 h). For each cross, 60 anthers were removed from each spike, and anthers from each side of the spike (30) were placed on separate sections of a 90 × 14 mm Petri dish containing mannitol pretreatment medium. Following anther removal, 20 ovaries were removed from each spike and placed in two 55 × 14 mm Petri dishes containing 4 mL LIM (10 ovaries per dish). All dishes were sealed with Parafilm™ and incubated in the dark at 25 °C for 5 days.

As per Experiment 1, anthers were exposed to caffeine immediately following mannitol and *n*-butanol pretreatment at the start of the induction phase. Following the removal of LIM plus *n*-butanol, anthers were either processed using the standard protocol (control) or treated with 0.5 mM caffeine in LIM, for 24 h, as per Experiment 1.

#### 4.3.2. Plant Regeneration and Grow-Out

Embryos and regenerant plants were processed and harvested in the same manner as Experiment 1 with minor modifications. Firstly, albino plants were not counted. Secondly, a different grow-out location was used in South Australia for two of the crosses (Trojan/Havoc and Trojan/Chief CL) due to limitations with greenhouse space at DPIRD South Perth (DPI). Due to the space constraints, other alternatives for regenerant plant grow-out were explored for our program. For this study, a small, specialized company that provides grow-out and seed-bulking services for cereals in Virginia (VIR), South Australia was used, and plants were transferred to this location. Ten to fifteen plants for each treatment/spike combination (400–600 plants per cross) were transplanted to soil. This was not possible for Scepter/05PN240, as green plant numbers were low for this cross, so only 233 regenerant plants were transplanted to soil (Table 3). Where possible, we attempted to balance plant numbers across treatments and spikes.

### 4.4. Experiment 3. The Effect of Trifluralin on Green and Albino Plant Regeneration and Chromosome Doubling

#### 4.4.1. Anther Culture

The application of trifluralin was tested on the variety Tammarin Rock. This variety was screened in an earlier study, in which it exhibited good green plant regeneration but a relatively low frequency of chromosome doubling (30%) [11]. Eight donor plants were sown over two sowing dates (Table 5). Spikes 1–10 were harvested from the first sowing date, and spikes 11–20 were harvested from the second sowing date. Spikes were harvested over 6 days and stored at 4 °C for up to 2 days. The experimental design and protocol was the same as Experiment 1 with anthers from spikes 1–10 exposed to three treatments (control, 1 µM trifluralin for 24 h and 1 µM trifluralin for 48 h) and anthers from spikes 11–20 exposed to different treatments (control, 3 µM trifluralin for 24 h and 3 µM trifluralin for 48 h) (Table 7). As per Experiment 1, anthers were exposed to trifluralin immediately following mannitol and *n*-butanol pretreatment at the start of the induction phase. Trifluralin (N13689, Sigma-Aldrich) was dissolved in sterile dimethyl sulfoxide (DMSO) (D2650, Sigma-Aldrich), and 10 or 30 µL of trifluralin stock was added to 4 mL LIM to provide final concentrations of 1 or 3 µM, respectively.

#### 4.4.2. Plant Regeneration and Grow-Out

Embryos and regenerant plants were processed in the same manner as Experiment 1 except that 10 plants per treatment/spike combination (100 plants per treatment) were transplanted to soil, where possible. Plant numbers were reduced slightly in the 3 µM trifluralin treatments (due to the negative effect of the treatments) and, as per Experiment 1, there were more plants transplanted from the control treatment, as a control was included in each set of 10 spikes.

### 4.5. Data Analysis

All analyses were performed using Genstat Edition 19 (http://genstat.com). The HGLM procedure was used to fit hierarchical generalized linear models for green and albino plants, and generalized linear models for % DH. A fixed effect of treatment was considered for all experiments. For Experiment 2, genotype and genotype by environment interactions (G*T) were also considered. Random effects of source spike were considered for all responses; these were not significant for % DH and not included in the reported models. Over-dispersion occurs when the data are more variable than the standard assumptions of a generalized linear model allow. This can be accounted for by allowing the dispersion parameter to be estimated as part of the modeling process. All models were run with and without (fixed value at 1) an estimation of the dispersion parameter. When the dispersion parameter was significant, outputs were reported from the model with an estimated dispersion parameter. *p*-values reported were from Wald tests for the dropping of fixed terms.

The numbers of green and albino plants were analyzed using a Poisson model. An identity link function was used to generate symmetrical LSDs.

For green plants in Experiment 2, genotype and G*T differences were explored using an iterative post-hoc analysis. At each stage, the genotype with the largest detected effect was omitted, and the G*T analysis was rerun. This was repeated until no significant genotype effects were identified. One genotype (Trojan/Havoc) was particularly responsive in terms of green plant production; removing it from the analysis enabled us to detect differences between treatments in another genotype. For albino plants in Experiment 3, one spike for Tammarin Rock was omitted from the analysis, as very low numbers of albino plants caused model fitting to fail.

The percentage (proportion) of DH plants was calculated as the total number of DHs/number of transplanted plants that survived to maturity × 100 and analyzed using a Binomial model with the identity link function. For Experiment 2, analyses were done separately for the two grow-out locations, as the locations differed for several variables, including pot size and fertilizer regime, and environments, including temperature and radiation.

Treatment means were compared using 5% least significant differences (LSDs). In one instance (Experiment 3, % DH), differences were only observed at the 10% level and a 10% LSD was reported. LSDs were calculated by multiplying the average standard error of difference (SED) by 2 (5% LSD) or 1.645 (10% LSD).

The success index for Experiments 1 and 3 was calculated for each treatment/spike combination by multiplying the number of green plants by % DH to obtain the number of DHs per 20 anthers. Values were then averaged for each treatment.

## Figures and Tables

**Table 1:** Means (± standard error [SE]) of green and albino plant numbers, expressed per 20 anthers, and chromosome doubling (% doubled haploid (DH)) following caffeine treatment during anther culture on the (Yr57/3*Gladius#60)/2*Trojan cross in 2017. LSD: least significant difference.

Treatment	Green Plants per 20 Anthers	Albino Plants per 20 Anthers	No. of Plants Transplanted	% DH
Control	38.6 (±3.42)	16.6 (±1.97)	100	56 (±5.0)
0.5 mM caffeine for 24 h	41.5 (±3.93)	17.0 (±2.33)	50	68 (±6.6)
0.5 mM caffeine for 48 h	24.3 (±3.68)	22.0 (±2.44)	50	54 (±7.1)
1.5 mM caffeine for 24 h	36.6 (±3.81)	11.0 (±2.10)	50	67 (±6.8)
1.5 mM caffeine for 48 h	27.5 (±3.67)	13.1 (±2.15)	45	67 (±7.0)
5% LSD	5.56	3.72		18.4

**Table 2:** Means (±SE) of green plant numbers, expressed per 30 anthers, following caffeine treatment during anther culture on five crosses in 2018.

Cross	Green Plants per 30 Anthers		
	Control	Caffeine (0.5 mM/24 h)	5% LSD
Trojan/Havoc	141.1 (±4.71)	161.0 (±4.88)	10.94
Lancer/LPB14-0392	32.8 (±2.14)	41.8 (±2.25)	5.21
Coolah/LPB16-3182	14.9 (±1.91)	14.4 (±1.90)	
Trojan/Chief CL	28.7 (±2.10)	25.3 (±2.05)	
Scepter/05PN240	5.9 (±1.81)	6.4 (±1.81)	

**Table 3:** Means (±SE) of chromosome doubling (% DH) following caffeine treatment during anther culture on five crosses in 2018. Plants were grown to maturity at DPIRD South Perth (DPI) or Virginia, South Australia (VIR). The number of plants transplanted for each treatment and cross is indicated in parentheses.

Cross	Grow-Out Location	% DH		
		Control	Caffeine (0.5 mM/24 h)	5% LSD
Lancer/LPB14-0392	DPI	31 (±2.6)	31 (±2.5)	*9.6*
		(309)	(351)	
Coolah/LPB16-3182	DPI	29 (±2.7)	35 (±3.0)	
		(285)	(249)	
Scepter/05PN240	DPI	62 (±4.4)	61 (±4.7)	
		(125)	(108)	
Trojan/Havoc	VIR	64 (±3.5)	58 (±3.6)	*10.0*
		(200)	(190)	
Trojan/Chief CL	VIR	47 (±3.6)	42 (±3.5)	
		(200)	(200)	

**Table 4:** Means (±SE) of green and albino plant numbers, expressed per 20 anthers, and chromosome doubling (% DH) following trifluralin treatment during anther culture on Tammarin Rock in 2018.

Treatment	Green Plants per 20 Anthers	Albino Plants per 20 Anthers	No. of Plants Transplanted	% DH
Control	31.8 (±1.73)	20.1 (±2.27)	225	38 (±3.3)
1 µM trifluralin for 24 h	25.1 (±2.15)	24.7 (±3.47)	100	49 (±5.1)
1 µM trifluralin for 48 h	20.0 (±2.02)	27.8 (±3.68)	100	51 (±5.1)
3 µM trifluralin for 24 h	10.8 (±1.77)	4.0 (±1.47)	97	46 (±5.3)
3 µM trifluralin for 48 h	9.2 (±1.72)	6.1 (±1.82)	78	53 (±5.8)
5% LSD	4.16	7.44		
10% LSD ^1^				11.5

^1^ A 10% LSD was used for % DH in this experiment as the omnibus *p*-value is between 0.05 and 0.1.

**Table 5:** Success index, expressed as DHs per 20 anthers, following caffeine treatment on the (Yr57/3*Gladius#60)/2*Trojan cross and trifluralin treatment on Tammarin Rock.

Caffeine Treatment	DHs per 20 Anthers	Trifluralin Treatment	DHs per 20 Anthers
Control	20.1	Control	10.7
0.5 mM caffeine for 24 h	29.5	1 µM trifluralin for 24 h	11.2
0.5 mM caffeine for 48 h	13.4	1 µM trifluralin for 48 h	9.7
1.5 mM caffeine for 24 h	25.5	3 µM trifluralin for 24 h	5.1
1.5 mM caffeine for 48 h	18.4	3 µM trifluralin for 48 h	4.6

**Table 6:** Spring wheat crosses/varieties used in this study.

Experiment	Cross or Variety	Sowing Date	No. of Donor Plants
Exp 1: The effect of caffeine on green and albino plant regeneration and chromosome doubling (one cross)	[Yr57/3*Gladius#60]/2*Trojan	3/3/17	3
Exp 2: The effect of caffeine on green plant regeneration and chromosome doubling (five crosses)	Trojan/Havoc	2/3/18	5
	Trojan/Chief CL	2/3/18	5
	Lancer/LPB14-0392	23/3/18	6
	Coolah/LPB16-3182	23/3/18	6
	Scepter/05PN240	6/4/18	6
Exp 3: The effect of trifluralin on green and albino plant pant regeneration and chromosome doubling	Tammarin Rock	13/7/18 & 27/7/18	8

**Table 7:** Caffeine and trifluralin treatments for Experiments 1 and 3. Anthers were exposed to the chemicals at the start of the induction phase.

Experiment	Spike Numbers	Treatment A	Treatment B	Treatment C
Experiment 1	Spikes 1–10	Control	0.5 mM caffeine for 24 h	0.5 mM caffeine for 48 h
	Spikes 11–20	Control	1.5 mM caffeine for 24 h	1.5 mM caffeine for 48 h
Experiment 3	Spikes 1–10	Control	1 µM trifluralin for 24 h	1 µM trifluralin for 48 h
	Spikes 11–20	Control	3 µM trifluralin for 24 h	3 µM trifluralin for 48 h
